# Impact of growth pH and glucose concentrations on the CodY regulatory network in *Streptococcus salivarius*

**DOI:** 10.1186/s12864-018-4781-z

**Published:** 2018-05-23

**Authors:** Jianing Geng, Szu-Chuan Huang, Yueh-Ying Chen, Cheng-Hsun Chiu, Songnian Hu, Yi-Ywan M. Chen

**Affiliations:** 1 Key Laboratory of Genome Sciences and Information, Beijing Institute of Genomics, Chinese Academy of Sciences, Beijing, China; 2 Department of Microbiology and Immunology, College of Medicine, Chang Gung University, Taoyuan, Taiwan; 3 Graduate Institute of Biomedical Sciences, College of Medicine, Chang Gung University, Taoyuan, Taiwan; 4 Molecular Infectious Disease Research Center, Chang Gung Memorial Hospital, Linkou, Taiwan

**Keywords:** *Streptococcus salivarius*, CodY, Growth pH, Carbohydrate availability, Transcriptome, Stress response, Virulence

## Abstract

**Background:**

*Streptococcus salivarius* is an abundant isolate of the human oral microbiota. Since both pH and glucose availability fluctuate frequently in the oral cavity, the goal of this study was to investigate regulation by CodY, a conserved pleiotropic regulator of Gram positive bacteria, in response to these two signals. The chemostat culture system was employed to precisely control the growth parameters, and the transcriptomes of wild-type *S. salivarius* 57.I and its CodY-null derivative (Δ*codY*) grown at pH 7 and 5.5, with limited and excessive glucose supply were determined.

**Results:**

The transcriptomic analysis revealed that CodY was most active at pH 7 under conditions of glucose limitation. Based on whether a CodY binding consensus could be located in the 5′ flanking region of the identified target, the transcriptomic analysis also found that CodY shaped the transcriptome via both direct and indirect regulation. Inactivation of *codY* reduced the glycolytic capacity and the viability of *S. salivarius* at pH 5.5 or in the presence of H_2_O_2_. Studies using the *Galleria mellonella* larva model showed that CodY was essential for the toxicity generated from *S. salivarius* infection, suggesting that CodY regulation was critical for immune evasion and systemic infections. Furthermore, the CodY-null mutant strain exhibited a clumping phenotype and reduced attachment in biofilm assays, suggesting that CodY also modulates cell wall metabolism. Finally, the expression of genes belonging to the CovR regulon was affected by *codY* inactivation, but CodY and CovR regulated these genes in opposite directions.

**Conclusions:**

Metabolic adaptation in response to nutrient availability and growth pH is tightly linked to stress responses and virulence expression in *S. salivarius*. The regulation of metabolism by CodY allows for the maximal utilization of available nutrients and ATP production. The counteractive regulation of the CovR regulon could fine tune the transcriptomes in response to environmental changes.

**Electronic supplementary material:**

The online version of this article (10.1186/s12864-018-4781-z) contains supplementary material, which is available to authorized users.

## Background

Global transcriptional regulators execute the co-regulation of multiple pathways simultaneously, which allows microbes to adapt quickly to environmental changes. CodY is one such conserved regulator that controls the expression of both metabolic and virulence genes in response to nutrient starvation in Gram positive bacteria with low GC content [1, 2]. CodY functions primarily as a repressor during exponential growth, and its repressive action is inhibited when nutrients become scarce [3]. The crystal structures of *Bacillus subtilis* CodY fragments show that CodY harbors a GAF domain at the N-terminal region which interacts with branched-chain amino acids (BCAAs, e.g. isoleucine, leucine, and valine) [4]. The binding of the effectors leads to a change in conformation and activation of the C-terminal helix-turn-helix DNA binding domain [4, 5]. Functional analysis indicates that both BCAAs and GTP could activate the DNA binding activity of CodY in *B. subtilis* [6–9], whereas BCAAs are the only known effectors for CodY in *Lactococcus lactis* [10], *Streptococcus mutans* [11], and *Streptococcus pneumoniae* [12]. A recent crystal structure study of full-length CodY demonstrates that GTP binds to a site located in the hinge region connecting the N- and C-terminal domains. In comparison to the CodY proteins that are activated by GTP, the CodY proteins of *L. lactis* and *S. pneumoniae* exhibit the substitution of three amino acids in the proposed GTP binding site, which may explain the specificity of the cofactors [13]. The CodY binding consensus sequence, 5’-AATTTTCWGAAAATT, has been defined in *B. subtilis* and *L. lactis* [14, 15].

Although CodY is activated by BCAAs and mainly acts as a repressor, *Listeria monocytogenes* CodY remains active in the absence of BCAAs. Under such conditions CodY can activate the expression of *prfA*, encoding a master virulence regulator [16], and subsequently initiate a multifaceted regulatory network. In *Bacillus thuringiensis*, CodY upregulates the PapR-PlcR quorum sensing system by optimizing the uptake of PapR, the signaling peptide of the transcriptional regulator PlcR. The surplus PapR activates the DNA binding activity of PlcR, and subsequently upregulates the expression of the PlcR regulon and virulence of *B. thuringiensis* [17]. The above examples demonstrate the diversity of CodY regulation.

*Streptococcus salivarius* is the primary ureolytic species in the human mouth. Since urea is the most abundant nitrogen source in the saliva and crevicular fluids [18], the presence of ureolytic *S. salivarius* is critical for maintaining the pH homeostasis of the oral ecosystem and preventing the development of dental caries [19]. Past studies revealed that the expression of *S. salivarius* 57.I urease operon was upregulated during growth at acidic pH values and in the presence of excess carbohydrate [20]. Under these conditions, ureolysis is beneficial for the survival of *S. salivarius* [21]. In vivo and in vitro analyses have confirmed that the repression of the urease operon at pH 7 with limited glucose supply is mainly governed by CodY [22]. It is intriguing that the activity of CodY is modulated by growth pH and carbohydrate availability in *S. salivarius*, as neither factor is known to affect CodY activation. This observation leads to the hypothesis that growth pH and carbohydrate availability modulate the regulatory activity of CodY by altering the intracellular metabolite profile. This study analyzed the transcriptomes of chemostat-grown *S. salivarius* 57.I and its CodY-null derivative (strain Δ*codY*) to construct the regulatory network of CodY in response to different growth conditions. The impact of CodY regulation on the stress responses and pathogenic capacity of *S. salivarius* was examined.

## Results

### The binding consensus sequence of CodY in streptococci

To define the CodY binding consensus in streptococcal species, the CodY regulons of *Streptococcus agalactiae*, *Streptococcus dysgalactiae subsp. equisimilis*, *Streptococcus gallolyticus* UCN34, *Streptococcus gordonii* CH1, *Streptococcus mitis* B6, *S. mutans* UA159, *S. pneumoniae* TIGR4, *Streptococcus pyogenes* M1, *Streptococcus sanguinis* SK36, *Streptococcus sui*s 05ZYH33, *Streptococcus thermophilus* CNRZ1066, and *Streptococcus uberis* 0140 J were downloaded from the database and their putative CodY binding consensus sequence was determined. The consensus sequence (Additional file 1: Figure S1A), 5’-AATTTTCAGAAAATT, is close to the known CodY binding consensus sequence (Additional file 1: Figure S1B).

### An overview of CodY-regulated genes and the role of CodY in global regulation

**Fig. 1:**
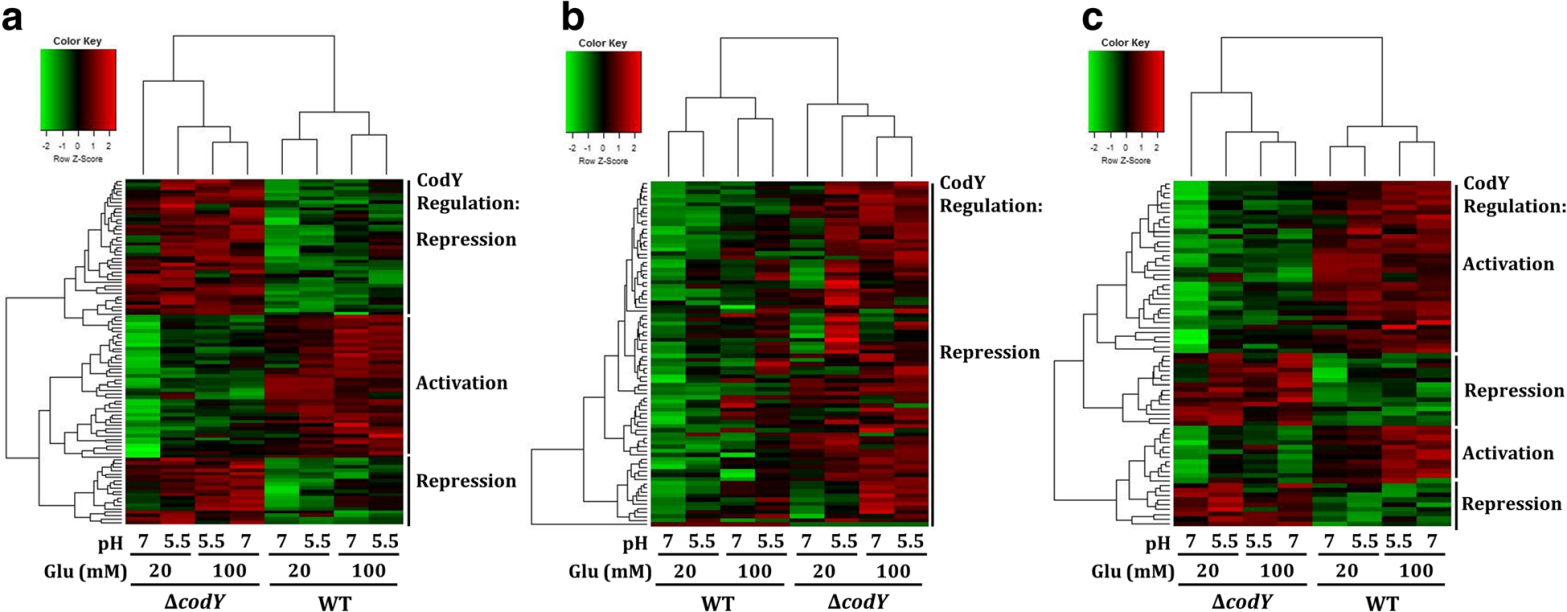
Hierarchical clustering of differentially transcribed genes in chemostat-grown *S. salivarius* 57.I and Δ*codY*. The expression patterns of differentially expressed genes under all growth conditions between the two strains (**a**), and the genes directly (**b**) and indirectly regulated by CodY (**c**) are shown. Bacterial cells were cultivated in TY containing 20 mM or 100 mM glucose, and the cultures were maintained at pH 7 or 5.5. The mode of CodY regulation is indicated on the right hand side of the figure

CodY regulates the expression of *S. salivarius* urease operon at both pH 7 and 5.5, with a limited (20 mM) or an excessive (100 mM) amount of glucose [22], suggesting that different levels of CodY activity would be detected in the growth conditions in this study. To test this hypothesis, the *codY* gene of *S. salivarius* 57.I was insertionally inactivated to generate strain Δ*codY.* Genes that are differentially expressed between wild-type 57.I and strain Δ*codY* under each growth condition were identified by using edgeR [23] and the regulation pattern of these genes was examined by using hierarchical clustering analysis (Fig. 1a). The expression patterns of wild-type 57.I and strain Δ*codY* under different growth conditions were located in two clusters, demonstrating the global impact of CodY regulation in response to growth pH values and glucose concentrations (Fig. 1a). Furthermore, the expression patterns of wild-type 57.I grown in 20 mM and 100 mM glucose were separated, suggesting that the amount of glucose in the culture medium modulates CodY activity more effectively than pH. Finally, the expression pattern of strain Δ*codY* under 20 mM glucose at pH 7 was most distant from that of the others, suggesting that CodY is most active at pH 7 with limited glucose supply.

As both activation and repression were observed (Fig. 1a), whether CodY affects the transcription profile of *S. salivarius* by both direct and indirect regulation was analyzed. Genes whose expression was affected significantly by *codY* deletion under at least one condition were identified. The direct targets were defined based on the presence of a CodY binding consensus in the 5′ flanking region of the gene or operon. A total of 153 genes was found; 83 and 70 genes are defined as direct and indirect targets of CodY, respectively. Hierarchical clustering analysis also indicated that the expression profile of strain Δ*codY* under 20 mM glucose at pH 7 was most distant from the profiles of the other three conditions, in both direct (Fig. 1b) and indirect targets (Fig. 1c). While all direct targets were upregulated in strain Δ*codY*, both upregulation and downregulation were observed in the indirect targets. The RPKM (Reads Per Kb per Million mapped reads) values of direct and indirect targets are listed in Additional file 2: Table S1 and S2, respectively. The targets are discussed below.

### Genes regulated directly by CodY

In agreement with observations in other bacteria, genes encoding proteins catalyzing the uptake and degradation of peptides, the uptake and biosynthesis of amino acids and urease were repressed directly by CodY in *S. salivarius* 57.I (Table 1). Although repression by CodY was generally more active under glucose limitation compared to that under excessive glucose, the effect of pH on the regulation of *oppA* (Ssal_00190), *pepC* (Ssal_00280) and *pepP* (Ssal_01946) was unclear. On the other hand, in most of the genes involved in the biosynthesis or uptake of amino acids, the expression level between wild-type 57.I and strain Δ*codY* was significantly different under at least three conditions, indicating that the differential expression of these genes in response to growth pH values and carbohydrate concentrations optimizes the fitness of *S. salivarius* under different growth conditions. Finally, all the genes of the urease operon were derepressed at pH 5.5 and upregulation was further enhanced under excessive glucose in *S. salivarius* 57.I (Additional file 2: Table S1). Additionally, the differences in the expression level between wild-type 57.I and strain Δ*codY* under glucose limitation were generally greater than that under excessive glucose, and the effect of *codY* deletion at pH 5.5 was less evident under excessive glucose, supporting the hypothesis that CodY is least active at pH 5.5 under excessive glucose.

**Table 1:** Genes directly regulated by CodY in *S. salivarius* 57.I

Function category/ Locus_tag	Gene/ Homolog^a^	Δ*codY*/57.I^b^				Position(nt)^c^	Predicated CodY binding sequence	Note^d^
		20 mM Glu		100 mM Glu				
		pH 7	pH 5.5	pH 7	pH 5.5			
Peptide uptake and degradation								
Ssal_00190	*oppA*	ns	ns	1.5	ns	94	AATATTCAGAAAGTA	
Ssal_00280	*pepP*	ns	4.3	ns	1.3	39	TAAAGTCTGAAAATT	
Ssal_01946	*pepC*	3.3	ns	ns	ns	42	AATTTTCAGAAATTA	
Amino acid uptake and biosynthesis								
Ssal_00127	*thrC*	1.6	ns	2.9	1.45	43	AATATTCTGACAATT	
Ssal_00131	*ilvD*	3.2	7.7	ns	3.1	66	AATATTCTGAAAATT	
Ssal_00132	*ilvB*	3.6	4.0	88.1	5.1	30	AATTTTTAGACAATT	Ssal_00132–00134 are predicted to be an operon
Ssal_00133	*ilvH*	5.1	6.3	4.4	2.4			
Ssal_00134	*ilvC*	5.3	9.7	2.4	1.6			
Ssal_00456	*–*	5.4	ns	ns	1.6	336	AATTAACAGAAAATT	Ssal_00456–00465 are predicted to be an operon
Ssal_00457	*trpE*	1.5	2.5	ns	1.6			
Ssal_00458	*trpG*	ns	3.5	ns	ns			
Ssal_00459	*trpD*	ns	2.3	ns	1.6			
Ssal_00461	*trpC*	ns	2.4	ns	ns			
Ssal_00462	*trpF*	ns	2.1	ns	ns			
Ssal_00464	*trpB*	ns	2.7	ns	ns			
Ssal_00465	*trpA*	ns	2.2	ns	1.5			
Ssal_00537	*serC*	2.0	2.6	2.5	2.6	38	AATATTCAGAAAATT	Ssal_00537–00539 are a predicted to be an operon
Ssal_00538	*–*	1.5	5.0	2.3	1.7			
Ssal_00539	*serA*	3.1	6.5	1.7	2.6			
Ssal_00884	*dapA*	1.8	ns	1.6	ns	74	CGTTTTCAGAAAATT	
Ssal_00898	*pdxU2*	2.6	3.3	2.8	3.8	41	AATCGTCAGAATTTT	Ssal_00898–00900 are predicted to be an operon
Ssal_00899	*pdxK*	4.6	4.6	2.8	3.3			
Ssal_00900	*pdxU*	3.9	2.6	4.3	3.1			
Ssal_01037	*ilvB*	3.6	5.3	1.8	ns	154	AAAATTCACAATCTT	
						129	GATTGTCTGAAAACT	
Ssal_01272	*leuD*	1.8	ns	ns	ns			Ssal_01276–01272 are predicted to be an operon
Ssal_01273	*leuC*	2.4	3.1	2.2	ns			
Ssal_01275	*leuB*	2.8	2.8	20.5	ns			
Ssal_01276	*leuA*	3.1	4.4	1.8	ns	242	AAAAGTCAGTCAATT	
Ssal_01351	*mprF*	1.8	2.9	3.5	1.6			Ssal_01352–01351 are predicted to be an operon
Ssal_01352	*–*	ns	2.7	23.2	ns	134	AATATTCTGACTCTT	
Ssal_01567	*ilvE*	5.8	3.6	11.1	4.2	75	AATTGTCAGAATTTT	
Ssal_01655	*pheP*	5.1	2.3	2.2	4.0	95	AAAATTCTGAATATT	
Ssal_01693	*thrB*	2.5	ns	ns	1.7			Ssal_01694–01693 are predicted to be an operon
Ssal_01694	*–*	1.8	1.8	ns	ns	98	AATTTTCAGTAAAAA	
						55	AATATTCTGTCAATT	
Ssal_01811	*lysC*	5.9	5.2	9.4	5.6	39	AATTGTCAGAATTTT	
Ssal_01812	*asnA*	3.3	2.1	ns	1.7	61	AATTGTTGGAAAATT	
						41	ATTTTTCTGAAAAAT	
Ssal_01828	*livF*	1.7	4.3	2.2	1.7			Ssal_01832–01828 are predicted to be an operon
Ssal_01829	*livG*	1.5	6.7	4.3	1.8			
Ssal_01830	*livM*	ns	3.5	36.1	1.3			
Ssal_01831	*livH*	ns	9.2	3.4	2.6			
Ssal_01832	*livK*	ns	12.5	5.5	2.0	192	ACTATTCTGATATTG	
						14	AATTGTCTGATAATT	
Ssal_01840	*metB*	ns	ns	5.0	ns	215	ATGTTTCAGGCACTT	
						62	TATTTTCAGAAAATA	
Ssal_02023	*ilvA*	3.1	3.3	ns	1.7	45	TTTTTTCTGAAAATT	
						17	AATAGTTTGAATATT	
Urease operon								
Ssal_01891	*ureO*	8.0	8.3	2.3	ns			Ssal_01903–01891 are predicted to be an operon Ssal_01903 and Ssal_01904 are in opposite orientations
Ssal_01892	*ureQ*	3.5	5.0	2.4	1.8			
Ssal_01894	*ureM*	2.0	3.3	2.2	1.6			
Ssal_01895	*ureD*	ns	6.3	1.7	ns			
Ssal_01896	*ureG*	ns	3.5	4.6	ns			
Ssal_01897	*ureF*	ns	4.3	2.0	ns			
Ssal_01898	*ureE*	ns	3.8	2.9	1.2			
Ssal_01900	*ureC*	4.1	2.5	ns	2.6			
Ssal_01901	*ureB*	ns	ns	1.9	ns			
Ssal_01902	*ureA*	ns	2.8	1.8	ns			
Ssal_01903	*ureI*	2.0	3.5	2.2	1.6	211	TATTGTCAGAAACAG	
						58	AAATTTCTGAAAATT	
Ssal_01904	*–*	6.1	2.0	12.2	4.3	334	AATTTTCAGAAATTT	
						181	CTGTTTCTGACAATA	
Other transporters								
Ssal_00845	*–*	2.5	2.0	57.1	13.8	69	TAATTTCAGAAAATT	
Ssal_00908	*–*	33.9	16.7	55.1	16.6	92	ATTTTTCAGAAAATA	
						60	ATTATTCCAACAATT	
						19	ATTATTCTGAAAATT	
Ssal_01154	*–*	ns	2.8	7.8	1.3	144	AATATTCGGAAAATA	
Ssal_01464	*–*	7.1	7.1	7.1	2.4			Ssal_01466–01464 are predicted to be an operon
Ssal_01465	*fhuC*	4.9	7.1	2.7	2.5			
Ssal_01466	*hmuU*	8.7	7.9	3.9	4.7	116	AATTTTCTGACATTA	
Regulation								
Ssal_00404	*codY*	nd	nd	nd	nd	116	AATTTTCAGACAATT	
Ssal_00555	*cidA*	2.2	ns	2.1	1.4	175	AAATATTTGACTATT	Ssal_00555–00556 are predicted to be an operon
						79	AATTTACTGAAAACT	
Ssal_00556	*cidB*	1.6	ns	3.2	1.7			
Ssal_00962	*–*	21.8	18.6	21.9	19.8	108	AAGTATCTGAAATAG	
						39	AATTTTCAGAATATT	
Ssal_01667	*glnB*	43.2	8.4	3.4	6.0			Ssal_01668–01667 are predicted to be an operon
Ssal_01668	*nrgA*	15.2	19.9	61.2	7.5	361	CTATTTCAGAAGTTT	
						292	GAGCTTCTGAAAATG	
						78	AATTATCAAAAAATT	
Ssal_01704	*–*	2.5	ns	9.1	2.1	10	AATTATCAGAAAAGG	
Ssal_01769	*nadR*	ns	ns	4.9	1.4	288	AATTTTCAGAATCTT	
						264	TATTTTCTGCTATTT	
Carbon metabolism								
Ssal_01359	*–*	2.4	3.1	4.1	3.4			Ssal_01360–01359 are predicted to be an operon
Ssal_01360	*gapN*	3.4	6.3	ns	1.5	74	AATTTTCTGAATAAT	
Ssal_01545	*–*	4.7	2.7	3.3	3.1			Ssal_01546–01545 are predicted to be an operon
Ssal_01546	*folD*	3.8	4.9	3.0	3.2	138	CATAATCAGAAAAAA	
						72	AAAATTTAGAAAATT	
Ssal_01768	*gdhA*	12.9	15.7	3.5	6.3	353	GCTTTCCTGATAATT	
						61	AAATAGCAGAAAATA	
Ssal_01810	*pgmB*	ns	ns	3.6	ns	75	AAAATTCTGACAATT	
Others								
Ssal_00112	*–*	3.5	2.3	4.4	1.4	44	AATCGTCTGAATATT	
Ssal_00214	*–*	ns	ns	ns	2.1	324	CATCTTCTGAAATTT	
Ssal_00216	*–*	3.3	2.1	2.8	ns	229	AAATTTCAGAAGATG	
Ssal_00672	*–*	72.1	247.7	73.6	41.8	178	AATTGTCAGACAACT	
						123	AATTGTCAGAATGTT	
Ssal_00714	*–*	ns	7.3	2.2	ns	108	ACTTTTCTAAAAATT	
Ssal_01153	*udg*	1.9	ns	ns	4.1	129	TATTTTCCGAATATT	
Ssal_01428	*lysM*	ns	3.9	ns	8.7	143	AAATGTCAGATAAAT	
Ssal_01429	*lysS*	ns	3.2	ns	1.215	69	ATTTATCTGACATTT	

^a^, −, no assigned gene name

^b^, The values are the RPKM of the locus in *S. salivarius* Δ*codY* divided by that in wild-type 57.I under the same growth condition. ns, no significant difference. nd, not determined

^c^, The distance (in nucleotides) of the predicted CodY binding consensus to the translation start site of the locus

^d^, Operons started with a lower tag number are transcribed from the minus strand

The expression of genes encoding proteins involved in regulation, metal ions transportation, carbon metabolism, virulence, and other cellular functions was also regulated by CodY (Table 1). Specifically, the expression of Ssal_00908, encoding a putative Mn^2+^/Fe^2+^ transporter, and of *hmuU-fhuC-*Ssal_01464, an operon encoding a putative ferric ATP-binding cassette transporter was repressed by CodY under all conditions, suggesting that CodY modulates metal homeostasis in *S. salivarius* 57.I. CodY also repressed the expression of *gdhA* (Ssal_01768), encoding glutamate dehydrogenase (GDH), and the *nrgA* (Ssal_01668)-*glnB* (Ssal_01667) operon, encoding an ammonium transporter and a PII-like nitrogen regulator, respectively. The repression of these genes was maximized under glucose limitation, indicating that CodY regulates nitrogen utilization in response to carbon availability. Furthermore, the expression of *pgmB* (Ssal_01810), encoding β-phosphoglucomutase for converting β-D-glucose-1-phosphate to glucose 6-phosphate, was up regulated at pH 7 under excessive glucose in strain Δ*codY*. Finally, the identification of *lysM* (Ssal_01428, encoding a peptidoglycan binding protein) and the *cidAB* operon (Ssal_00555 and Ssal_00556, encoding a putative murein hydrolase exporter and a regulator) suggests that CodY regulates cell wall biosynthesis.

### Indirect targets of CodY

Forty-eight indirect targets were downregulated in strain Δ*codY* under at least 3 growth conditions, while 22 genes were upregulated (Table 2). Among the upregulated genes, Ssal_00630, Ssal_00982 and Ssal_01876 are annotated in GenBank as transcriptional regulators. Particularly, the protein product of Ssal_01876, CovR, is a dominant global regulator controlling virulence expression in streptococci [24–26]. Sequence analysis revealed that 33 of the indirect targets harbor a CovR binding consensus sequence (5′-ATTARA) [27] at the 5′ flanking region, and both upregulation (11 genes) and downregulation (22 genes) were observed in strain Δ*codY*. Specifically, a putative cell wall-binding hydrolase gene (Ssal_00236) was downregulated in Δ*codY*, suggesting that CodY indirectly modulates cell wall structure.

**Table 2:** Genes indirectly regulated by CodY in *S. salivarius* 57.I

Function category/ Locus_tag	Gene/ Homolog^a^	Δ*codY*/57.I^b^				Note^c^
		20 mM Glu		100 mM Glu		
		pH 7	pH 5.5	pH 7	pH 5.5	
Genes/operons with a CovR binding consensus						
Ssal_00236	*–*	0.01	0.005	0.01	0.01	
Ssal_00563	*–*	0.05	0.04	0.07	0.11	
Ssal_00678	*–*	0.00	0.39	0.21	0.07	
Ssal_00697	*–*	0.04	0.28	0.31	0.18	
Ssal_00723	*–*	0.03	0.01	0.12	0.05	
Ssal_00725	–	0.03	0.08	0.21	ns	
Ssal_00726	–	ns	0.15	0.30	ns	
Ssal_00728	–	0.01	0.03	0.22	0.25	
Ssal_00729	–	0.003	0.04	0.13	0.21	
Ssal_00732	–	0.08	0.41	0.03	0.32	Ssal_00733–00732 are predicted to be an operon
Ssal_00733	–	0.01	0.47	0.02	0.12	
Ssal_00734	–	0.005	0.03	0.07	0.11	Ssal_00737–00734 are predicted to be an operon
Ssal_00735	–	0.001	0.03	0.11	0.15	
Ssal_00736	–	0.001	0.02	0.05	0.16	
Ssal_00737	–	0.000	0.03	0.03	0.12	
Ssal_00755	–	0.004	0.02	0.03	0.30	
Ssal_00854	*–*	0.03	0.35	0.20	0.02	
Ssal_00871	*sacC*	0.01	0.01	0.01	0.13	
Ssal_01340	*–*	0.06	0.30	0.05	0.18	
Ssal_01662	*–*	0.06	0.10	0.12	0.12	
Ssal_01837	*sacB*	0.01	0.01	0.05	0.08	
Ssal_01861	*manH*	0.18	0.25	0.31	0.23	
Ssal_00446	*–*	9.2	2.7	10.7	1.9	
Ssal_00630	*gntR*	1.9	2.2	1.7	3.5	
Ssal_00969	*–*	8.9	14.7	2.4	5.4	
Ssal_01025	*–*	3.1	16.7	8.3	1.8	
Ssal_01086	*mnmE*	2.6	3.9	3.4	1.9	
Ssal_01401	*endA*	3.7	7.4	5.0	7.1	
Ssal_01479	*–*	5.2	2.9	19.1	5.3	
Ssal_01624	*nagB*	2.2	1.9	3.8	1.3	
Ssal_01876	*covR*	8.8	6.5	3.6	5.7	
Ssal_02041	*tig*	1.6	7.3	2.6	1.6	
Ssal_02096	*–*	3.7	2.8	6.0	3.1	
Others						
Ssal_00088	*rpsS*	0.04	0.48	0.16	0.22	
Ssal_00221	*rpe*	0.28	0.44	0.17	0.27	
Ssal_00318	*–*	0.13	0.24	0.36	0.18	
Ssal_00689	*–*	0.11	0.48	0.32	0.23	
Ssal_00700	*–*	0.06	0.35	0.14	0.10	
Ssal_00703	*–*	0.06	0.34	0.24	0.19	
Ssal_00750	*ece1*	0.34	0.47	0.21	0.32	
Ssal_00859	*–*	0.18	0.40	0.36	0.25	
Ssal_01403	*–*	0.00	0.02	0.29	0.02	
Ssal_01497	*–*	0.33	0.25	0.36	0.06	
Ssal_01636	*macB*	0.12	0.37	0.20	0.06	
Ssal_01796	*accA*	0.23	ns	0.14	0.16	Ssal_01807–01796 are predicted to be an operon
Ssal_01797	*accD*	0.13	0.40	0.09	0.16	
Ssal_01798	*accC*	0.12	ns	ns1	0.20	
Ssal_01799	*–*	0.11	ns	0.05	0.07	
Ssal_01800	*fabZ*	0.27	ns	0.05	0.21	
Ssal_01801	*accB*	ns	ns	0.22	ns	
Ssal_01802	*fabF*	0.16	0.39	0.05	0.15	
Ssal_01803	*fabG*	0.10	ns	0.12	0.14	
Ssal_01804	*fabD*	0.09	0.38	0.12	0.16	
Ssal_01805	*fabK*	0.06	ns	0.04	0.16	
Ssal_01806	*acpP*	0.31	0.36	0.16	ns	
Ssal_01807	*fabH*	0.25	0.50	0.17	0.20	
Ssal_01838	*–*	0.21	0.07	0.15	0.19	
Ssal_02030	*cpdB*	0.01	0.07	0.12	0.10	
Ssal_02089	*cysE*	0.26	0.43	0.10	0.16	
Ssal_00154	*–*	8.3	4.5	ns	2.7	
Ssal_00402	*–*	1.5	2.0	3.6	1.6	
Ssal_00485	*yuaJ*	4.6	5.3	6.5	2.7	
Ssal_00506	*–*	2.2	2.3	2.4	1.5	
Ssal_00665	*–*	3.5	2.2	2.4	2.9	
Ssal_00717	*mucB*	6	27.3	4.9	3.2	
Ssal_00738	*–*	50.1	193.2	28.3	4.0	
Ssal_00740	*–*	30.7	75.6	6.7	17.9	
Ssal_00742	*–*	3.6	13.2	1.8	1.2	
Ssal_00975	*–*	9.9	5.4	9.4	1.5	
Ssal_00982	*–*	1.9	1.9	1.7	1.2	

^a^, −, no assigned gene name

^b^, The values are the RPKM of the locus in *S. salivarius* Δ*codY* divided by that in wild-type 57.I under the same growth condition. ns, no significant difference

^c^, Operons started with a lower tag number are transcribed from the minus strand

Inactivation of CodY also led to the down-expression of a gene cluster (*fabH-acpP-fabKDGF-accB-fabZ-*Ssal_01799-*accCDA*) encoding proteins involved in membrane fatty acid biosynthesis. Although the effects of growth pH values and glucose concentrations on the regulation of these genes are unclear, the differences between wild-type 57.I and Δ*codY* in cells grown at pH 7 were generally greater than in those grown at pH 5.5 under the same concentration of glucose, suggesting that CodY was essential for optimal membrane biosynthesis at pH 7.

### Inactivation of CodY promoted clumping, prolonged the doubling time, and reduced the biofilm formation in *S. salivarius* 57.I

**Fig. 2:**
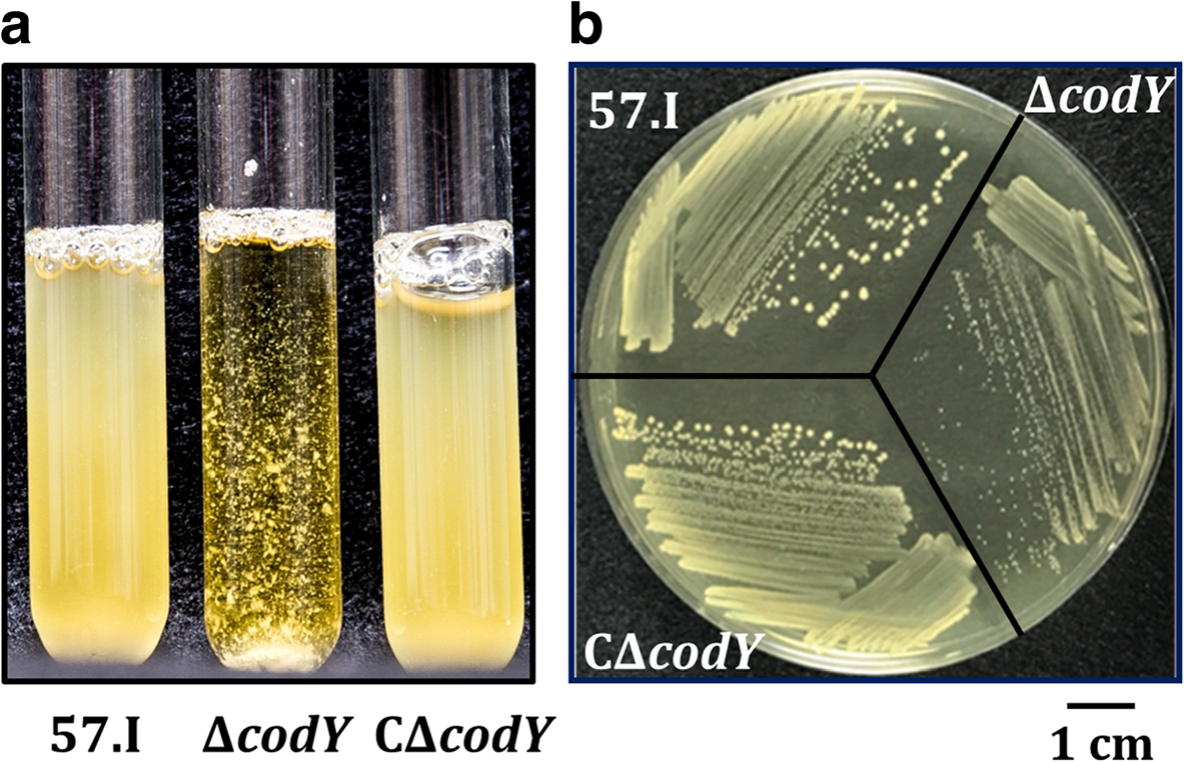
The phenotypic characteristics of *S. salivarius* 57.I and its derivatives. **a** Broth cultures of strains 57.I, Δ*codY* and CΔ*codY* in BHI. **b** The colonies on BHI agar

**Fig. 3:**
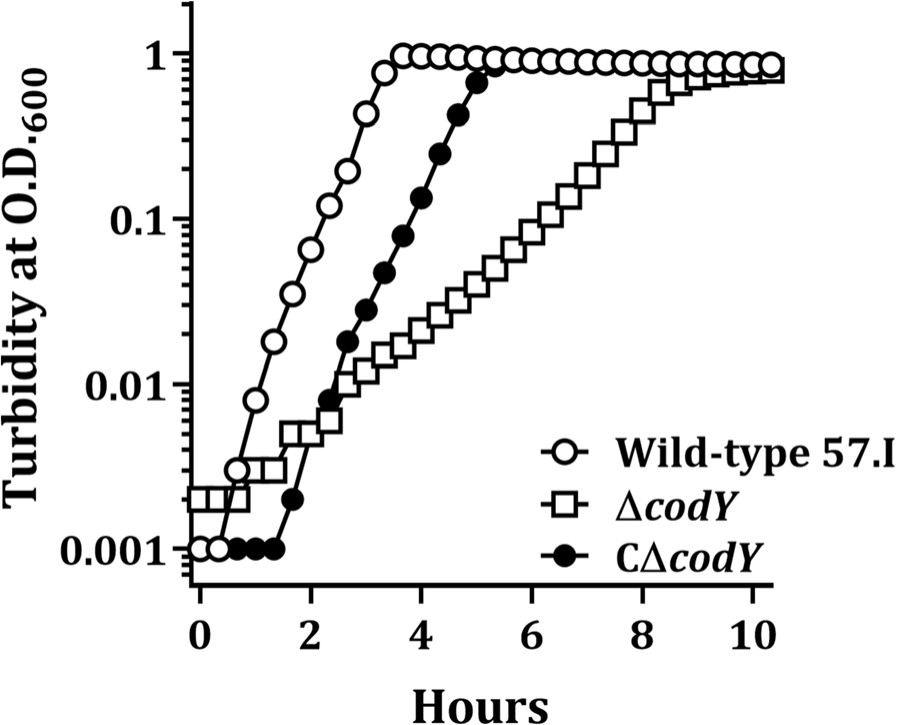
Growth kinetics of *S. salivarius* 57.I and its derivatives in BHI. The results are representative of those from three independent experiments

To analyze the impact of CodY regulation, a *codY* complemented strain, CΔ*codY*, was generated as detailed in the methods, in which a single copy of *codY* was established on the chromosome of strain Δ*codY*. During the preparation of *S. salivarius* cultures for growth studies, strain Δ*codY* formed aggregates in overnight cultures, while wild-type 57.I did not (Fig. 2a). The colony size of strain Δ*codY* was smaller than that of the wild-type 57.I (Fig. 2b). To reduce clumping during the course of the growth kinetic studies, the inocula of all strains were gently sonicated prior to inoculation and the cultures were shaken before each reading. The estimated generation time of wild-type 57.I in BHI was approximately 22 min, whereas strain Δ*codY* exhibited a generation time of approximately 44 min (Fig. 3). A wild-type doubling time was observed in the complemented strain (CΔ*codY*), although the lag phase in strain CΔ*codY* was longer than that of wild-type 57.I under the experimental condition for unknown reasons.

**Fig. 4:**
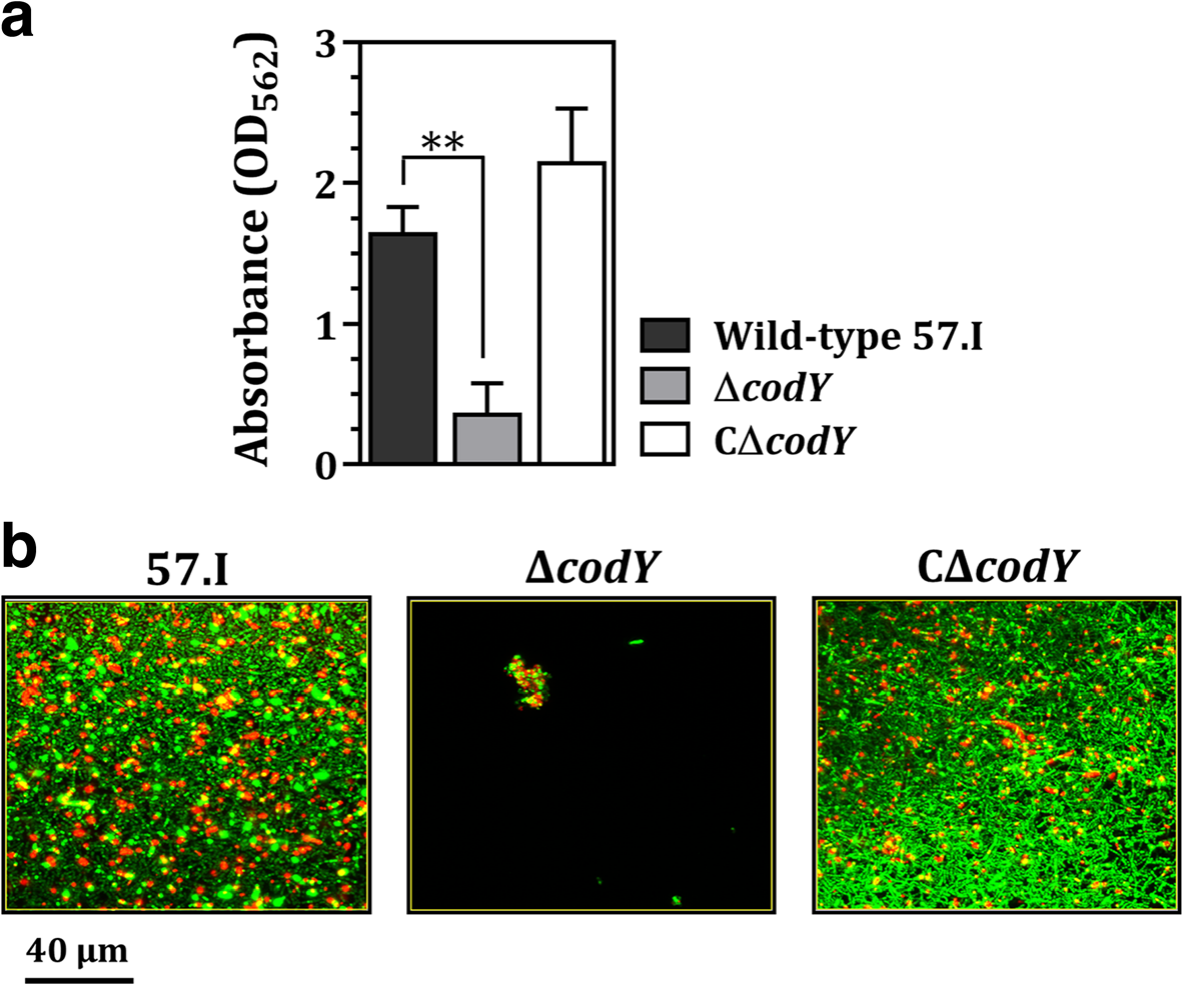
The biofilm formation by *S. salivarius* 57.I and its derivatives. **a** The mass of the static biofilms of *S. salivarius* 57.I, Δ*codY* and CΔ*codY*. Values are expressed in terms of the means and standard deviations of three independent experiments; quadruplicate samples of each strain were used in each experiment. Significant differences were analyzed by the Student’s *t* test. **, *p* < 0.01. **b** Images of the biofilms in the flow cell system. The biofilms were grown in BMG for 10 h. Live (green fluorescence) and dead (red fluorescence) cells were stained with SYTO9 and PI, respectively, and visualized by CLSM

In both the static biofilm cultures (Fig. 4a) and the flow-cell system (Fig. 4b), cells of strain Δ*codY* failed to adhere to the plastic surface effectively. Presumably, self-aggregation impeded colonization, leading to minimal biofilm formation. Both wild-type 57.I and strain CΔ*codY* formed thick biofilms containing both live and dead cells (Fig. 4b).

### Inactivation of CodY reduced the glycolytic rate, acid tolerance and the capacity against oxidative stress in *S. salivarius* 57.I

**Fig. 5:**
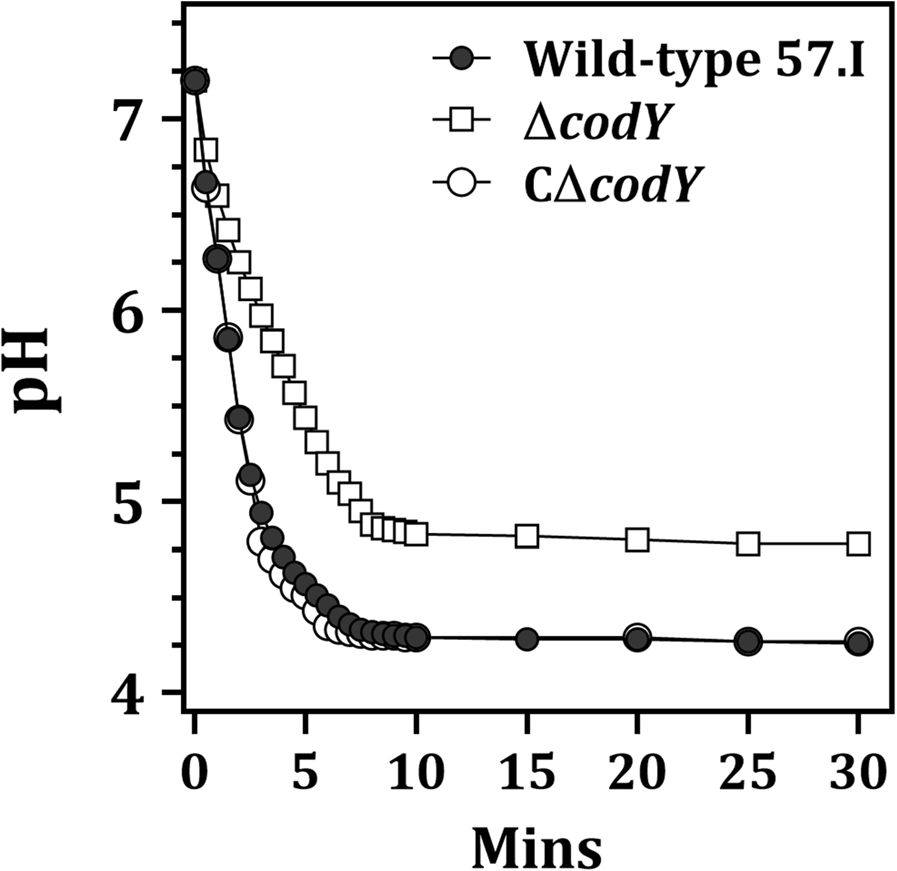
The acid production by *S. salivarius* and its derivatives. The cell suspensions at 10^9^ CFU ml^− 1^ were adjusted to pH 7.2 and glycolysis was initiated by the addition of 55.5 mM glucose. The results are representative of those from three independent experiments

**Fig. 6:**
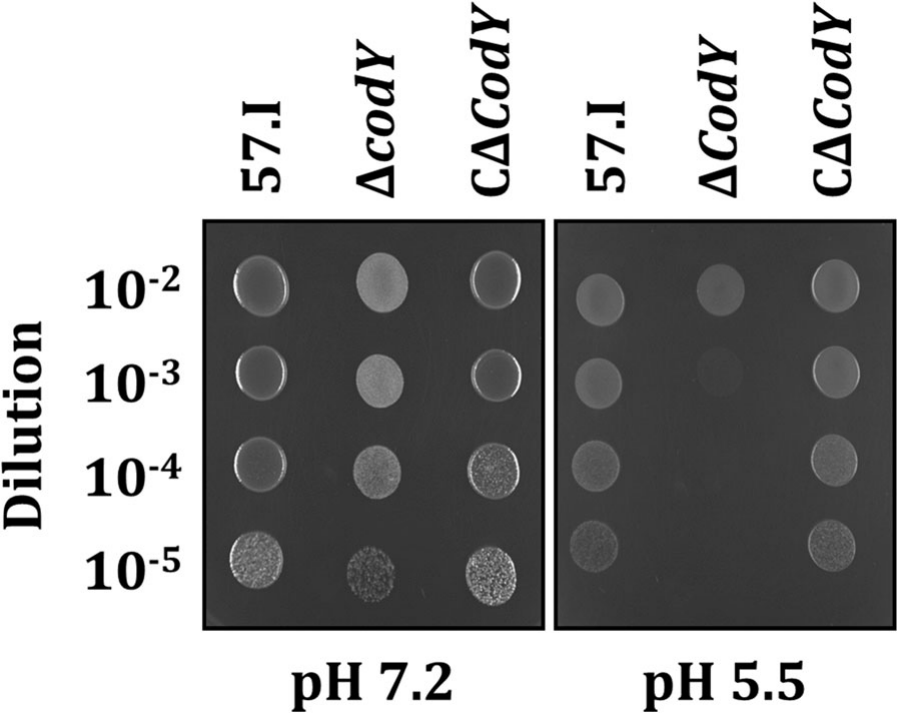
The effect of pH on the growth of *S. salivarius* strains. 10 μl of serially diluted exponential growth phase cultures of strains 57.I, Δ*codY*, and CΔ*codY* were spotted on BHI (pH 7.2) or BHI-HCl (pH 5.5) agar, and were grown for 24 h. The images are representative of three independent experiments

Regulation by CodY is likely to play a critical role in energy conservation, which could then influence acid tolerance via the activity of F-ATPase [28]. Since bacterial glycolysis is normally acid-limited, and the terminal pH value reflects the sensitivity of cells to acidification, the acid production from glucose and acid tolerance of all strains were evaluated to test this hypothesis. The acid production of strain Δ*codY* was lower than that of wild-type 57.I and strain CΔ*codY*, and the final pH of strain Δ*codY* was 0.5 unit higher than that of wild-type 57.I (pH 4.78 vs. pH 4.26) and strain CΔ*codY* (Fig. 5), suggesting that the acid tolerance of strain Δ*codY* was lower than that of wild-type 57.I. Additionally, the expression levels of *glk* (Ssal_01383), *pfk* (Ssal_01270) *pyk* (Ssal_01268), and *pgk* (Ssal_00232), encoding glucose kinase, 6-phosphofructokinase, pyruvate kinase and phosphoglycerate kinase in the Embden-Meyerhof-Parnas pathway, in wild-type 57.I were higher than those in strain Δ*codY* under at least three growth conditions (Additional file 2: Table S3), indicating that CodY positively modulates the glycolytic activity via an unknown mechanism. Finally, the growth of *S. salivarius* at pH 5.5 was compromised significantly by *codY* deletion (Fig. 6).

**Fig. 7:**
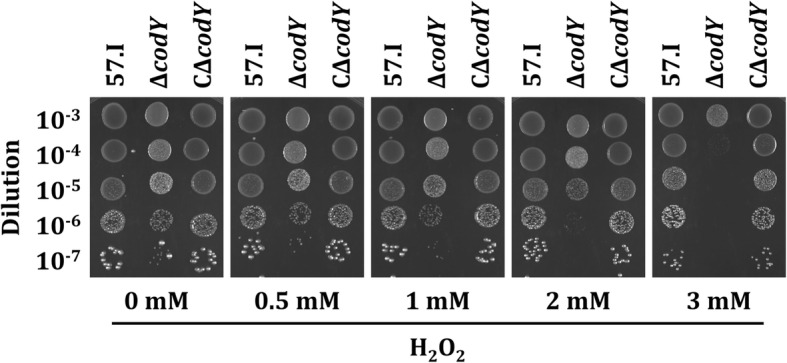
The effect of H_2_O_2_ on the growth of *S. salivarius* strains. 10 μl of serially diluted exponential growth phase cultures of strains 57.I, Δ*codY*, and CΔ*codY* were spotted on BHI agar containing various concentrations of H_2_O_2_ and were grown for 24 h. The images are representative of three independent experiments

CodY also participates in the regulation of the oxidative stress response. A comparable level of growth was observed in wild-type 57.I and strain CΔ*codY* in medium containing up to 3 mM H_2_O_2_, while reduction in growth was detected with strain Δ*codY* (Fig. 7), indicating that inactivation of CodY sensitized *S. salivarius* to oxidative stress.

### CodY regulation was essential for the toxicity generated from *S. salivarius* infection in the *G. mellonella* larva model

**Fig. 8:**
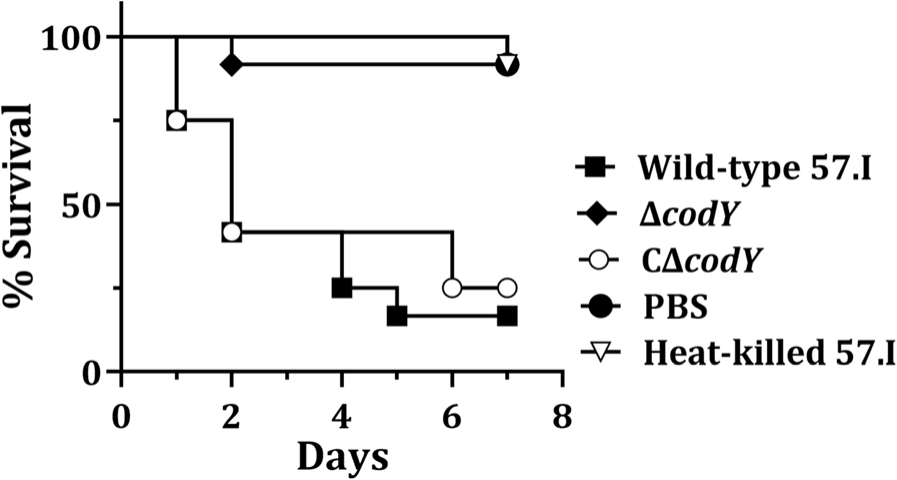
The toxicity of *S. salivarius* 57.I, Δ*codY* and CΔ*codY* in the *G. mellonella* larva model. Heat inactivated *S. salivarius* 57.I suspension and PBS were used as negative controls. 12 larvae were used for each bacterial strain. Kaplan-Meier survival analysis showing significant reduction of toxicity in *S. salivarius* Δ*codY* (*p <* 0.001). The results are representative of those from three independent experiments

To analyze whether *codY* deletion would affect *S. salivarius* against host innate immune clearance, the toxicity of *S. salivarius* was evaluated in the *G. mellonella* larva model (Fig. 8). Seven out of 12 larvae infected with *S. salivarius* 57.I died 2 days post infection, and greater than 10^7^ CFUs were recovered from one dead larva. A similar death rate was observed with the group of larva infected with strain CΔ*codY*. Less than 10^2^ CFUs was recovered from one live larva infected with strain Δ*codY* 7 days post infection. Thus, CodY regulation was crucial for the survival of *S. salivarius* and the death of the larvae.

## Discussion

Microbes in the oral cavity frequently encounter fluctuations in carbohydrate availability and pH. This study was designed to gain insight into the impact of these two environmental factors on CodY regulation. Using the chemostat culture system, we found that CodY is most active under glucose limitation. Reduced CodY repression under excessive glucose, when the level of nitrogen containing nutrients is likely to be limiting, could enhance the overall nutrient utilization and energy generation by promoting ammonium uptake and amino acid biosynthesis. Specifically, *S. salivarius* is able to utilize ammonium as a sole nitrogen source [21], therefore the expression of the *nrgA-glnB* operon under excessive glucose allows for the utilization of an alternative nitrogen source. In *S. thermophilus* [29], GDH mediates the conversion of carbon nutrients into amino acids to promote lactose utilization. Conceivably, the reduced repression of *gdhA* by CodY in *S. salivarius* under excessive glucose might promote the utilization of carbon nutrients by the upregulation of glutamate biosynthesis. On the other hand, variations in the effects of pH between CodY-regulated genes may result from additional pH-dependent regulation by other regulator(s), as seen in the regulation of the urease operon [22, 30]. Taken together, the current data suggest that the profile of intracellular metabolites, rather than the growth pH plays major roles in modulating CodY activity in *S. salivarius*.

In *S. pyogenes*, CovR is a master regulator of the CovRS two-component system, which predominantly acts as a transcriptional repressor [25]. Among the identified targets that potentially belong to the CovR regulon, some are activated by CodY in *S. salivarius*, suggesting that CodY and CovR regulate common genes in opposite directions. Similar regulation has been reported in *S. pyogenes*, in which CodY modulates CovRS-regulated transcriptomes in response to nutritional conditions [31]. The opposite regulation in response to specific growth conditions may fine tune the transcriptomes to enhance the fitness and virulence of *S. salivarius*. Thus, regulation by CovR in the absence of CodY modulation contributes, at least in part, to some of the phenotypes of Δ*codY*.

The clumping of strain Δ*codY* cells in liquid culture, which has not been reported in other CodY-null bacterial strains, is likely to result from CovR regulation in the absence of CodY modulation. It is established that defects in enzymes catalyzing cell wall metabolism could alter cell morphology, the chain length of streptococci [32], or the cluster size of staphylococci [33]. Among CodY-regulated genes that potentially participate in cell wall metabolism in *S. salivarius* 57.I, the expression of Ssal_00236, a potential member of the CovR regulon, is most affected by CodY deletion. Ssal_00236 is highly expressed in *S. salivarius* 57.I, the expression level of which is approximately 3–8 fold higher than that of *dnaA* under the same growth conditions (data not shown). Inactivation of CodY almost abolished the expression of Ssal_00236. Although the function of the protein encoded by Ssal_00236 and its homologs has not been characterized experimentally in streptococci, this protein is predicted to harbor a LysM motif and a CHAP domain in a manner similar to the Cse protein of *S. thermophilus* [34]. Cse interacts with the cell wall via the LysM motif and catalyzes cell wall hydrolysis via the endopeptidase activity of the CHAP domain. Inactivation of this locus results in abnormal daughter cell segregation and long chains of cells. The indirct regulation of Ssal_00236 by CodY might be required for the development of proper chain morphology. While the function of the CidAB system in cell wall hydrolysis has been demonstrated in both *S. mutans* [35] and *S. aureus* [36], in both cases the hydrolytic activity of the CidAB system is counteracted by the activity of the LrgAB system in response to the growth conditions, and neither system is regulated by CodY. Thus, the contribution of the up-expression of the *cidAB* operon and *lysM* (Ssal_01428) in the clumping of Δ*codY* cells is unclear.

It is likely that CodY participates in acid tolerance of *S. salivarius* 57.I by regulating the metabolic status, urease production, and possibly, membrane integrity. Regulation of metabolic pathways by CodY could augment ATP generation for acid tolerance, as seen in non-ureolytic *S. mutans* [11, 37–39]. Additionally, the regulation of the urease operon by CodY not only contributes to pH homeostasis but also provides a surplus of nitrogen-containing nutrients, which could again, promote ATP generation for pumping intracellular H^+^ via the activity of F-ATPase at acidic growth pH values. Finally, studies in *S. mutans* have demonstrated that the upregulation of unsaturated membrane fatty acid biosynthesis enhances acid tolerance [40, 41], indicating that membrane integrity is essential for optimal acid resistance. Thus, the down regulation of the *fabH-acpP-fabKDGF-accB-fabZ-*Ssal_01799-*accCDA* gene cluster in strain Δ*codY* could potentially lead to reduced membrane integrity and acid tolerance in *S. salivarius*.

CodY participates in the oxidative stress response in *S. thermophilus* [42] and *S. pneumoniae* [43] by activating the expression of genes encoding glutathione synthetase (*gshF*) and thiol peroxidase (*tpxD*), respectively. However, neither *gshA* (Ssal_00663, an ortholog of *gshF*) nor *tpx* (Ssal_01091) is regulated by CodY in *S. salivarius* 57.I. Instead, CodY regulates directly two iron uptake systems (the products of Ssal_00908 and *hmuU-fhuC-*Ssal_01464). Intracellular iron homeostasis is critical for controlling the generation of reactive oxygen species resulting from the Fenton reaction. In *S. pyogenes*, both upregulation of an Fe(II) exporter (PmtA) [44] or downregulation of iron uptake [45] enhances the oxidative stress resistance. Thus, it is possible that the elevated iron uptake in strain Δ*codY* could lead to the perturbation of iron homeostasis, leading to reduced resistance against H_2_O_2_.

A putative fibronectin-binding protein (FBP) (encoded by Ssal_00672) was more actively expressed in strain Δ*codY* than in wild-type 57.I under all growth conditions. Although homologs of FBP in Gram positive pathogens are known to participate in adherence, invasion and immune evasion [46, 47], the toxicity of strain Δ*codY* in the *G. mellonella* infection model was significantly lower than that of wild-type 57.I. Thus, either Ssal_00672 is not required for the survival against innate immune responses, or the upregulation of Ssal_00672 enhances recognition by host immune cells, and subsequently enhances clearance.

## Conclusions

**Fig. 9:**
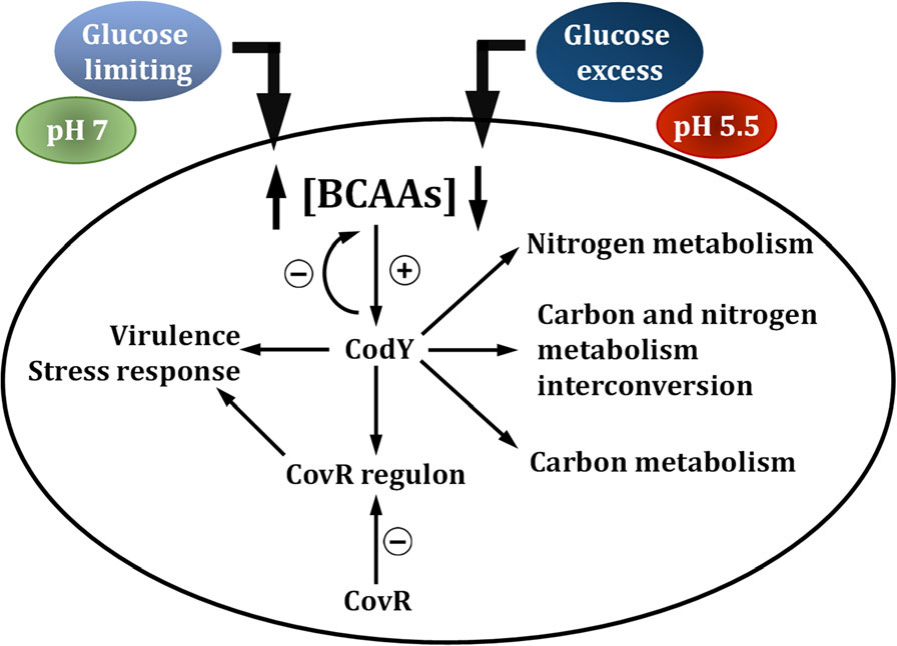
The regulation circuit of CodY in *S. salivarius*. The model suggests that the activity of CodY is modulated by growth pH and glucose concentrations via the amount of intracellular BCAA produced under each growth condition. Positive regulation is indicated by a plus sign and repression is indicated by a minus sign

In conclusion, this study demonstrated that metabolic adaptation in response to nutrient availability and growth pH is tightly linked to stress responses and virulence expression in *S. salivarius* (Fig. 9). The regulation of metabolism by CodY allows for the maximal utilization of available nutrients and ATP production. The counteractive regulation of the CovR regulon fine tunes the expression of the regulon in response to the nutrient availability and growth pH.

## Methods

### Construction of a *codY-*deficient and a *codY*-complemented derivative of *S. salivarius* 57.I

Primers used in this study are listed in Additional file 2: Table S5. Plasmid pSky2 [22], in which the region encoding the 25th to 78th amino acids (aa) of CodY was replaced by an erythromycin (Em) resistance gene (*erm*) [48], was used to insertionally inactivate *codY* in *S. salivarius* 57.I. The genotype of the Em resistant isolates was verified by colony PCR using primers Ssal_00403_S and Ssal_00405_AS. The correct strain was designated Δ*codY*. An Ω*kan*-tagged *codY* gene was then inserted into the intergenic region between Ssal_00777 and Ssal_00779 of strain Δ*codY* using ligation mutagenesis [49] to generate a *codY* complemented strain. A 1.1-kbp DNA fragment containing the *codY* gene, its 5′ flanking region of 250 bp and its 3′ flanking region of 90 bp was generated from *S. salivarius* 57.I with primers 57.I_CodY_800_XhoI_S and 57.I_CodY_1900_SphI_AS. A DNA fragment containing the Ω*kan* cassette was generated from plasmid pVT924 [50] with primers kan_BamHI_S and kan_XhoI_AS. Two DNA fragments for integration onto the chromosome were generated from *S. salivarius* 57.I using primer pairs 57.I_lacZ_1241_S/57.I_lacZ_4970_BamHI_AS and 57.I_lacZ_4970_SphI_S/57.I_ssal_00779_5950_AS, respectively. All four PCR products were digested, mixed in a ligation reaction and then used to transform *S. salivarius* Δ*codY* by natural transformation [51]. The genotype of the kanamycin resistant isolates was verified by colony PCR. The correct strain was designated CΔ*codY*.

### Bacterial strains and growth conditions

Bacterial strains used in this study are listed in Additional file 2: Table S4. *S. salivarius* 57.I [52] and Δ*codY* were grown in a chemostat culture system (Sartorius) in 3% tryptone-0.5% yeast extract medium (TY) supplemented with 20 mM or 100 mM glucose at a dilution rate of 0.3 h^− 1^, corresponding to a generation time of 2.3 h. Cultures were maintained at pH 7 or pH 5.5 by the addition of 2 N KOH. Cells were grown for a minimum of 10 generations to achieve the steady state at each condition.

### RNA-seq library construction, sequencing and gene expression analysis

Total cellular RNA was isolated from streptococcal strains as described [53] and further purified using the RNeasy kit (Qiagen). Ribosomal RNA was removed from the RNA preparation using the RiboMinus transcriptome isolation kits (Invitrogen). The RNA-seq library was constructed using SOLiD™ small RNA expression kit (ABI). All procedures were performed per manufacturer’s instruction. The sequence of the sample was determined on an ABI SOLiD 4.0 sequencer. Reads with a quality value greater than 8 were mapped to the genome sequence of *S. salivarius* 57.I (accession number CP002888.1) by using the SOLiD system analysis pipeline tool (Corona Lite) with mismatches up to 5 bases. RPKM were used to calculate the expression level of each gene. The RPKM values were log_10_ transformed and the correlation between biological and technical replicates under the same growth condition was examined to verify the quality of the analysis. The significant difference in the expression level of each gene between wild-type 57.I and strain Δ*codY* under one specific growth condition was analyzed using a Bioconductor software package edgeR with a *p*-value cut off at 0.05 [23].

### Identification and characterization of the targets of CodY

Information on the CodY regulons of various streptococcal species was retrieved from RegPrecise version 3.0 database [54]. A position weight matrix (PWM) of the 5′ flanking regions of the CodY regulon was constructed with the MEME software at the default parameter settings. The PWM model was used to search for potential CodY binding motif in the 400-bp region 5′ to the translation start site of all genes in *S. salivarius* 57.I by using MAST with a *p* value less than 10^− 4^ [55]. The transcriptomes of wild-type 57.I and strain Δ*codY* under each growth condition were used to seek targets of CodY by using the following criteria. Firstly, a significant difference in the expression level was observed between strains in at least one growth condition, and the regulation pattern (repression or activation) was identical in all conditions. Secondly, the regulation by CodY was consistent in genes of one operon. Thirdly, genes that are differentially expressed between strains and are with a CodY binding consensus were considered direct targets; genes without a CodY binding consensus were considered indirect targets.

### Growth kinetics

The growth kinetics of *S. salivarius* strains was analyzed using the Bioscreen C Microbiology Reader (Oy Growth Curve AB Ltd.) as described [56]. Overnight cultures were diluted at 1:20 in brain heart infusion (BHI) and grown to an optical density at 600 nm (OD_600_) of 0.8. The culture was sonicated at 10% amplitude twice, 5 s each, diluted at 1:1000 in BHI and then 300 μl of the diluted suspension was inoculated in a microtiter plate. The OD_600_ value was monitored at 10-min intervals for 10 h. The plate was shaken for 10 s before each reading.

### The static and the flow-cell biofilm systems

The static biofilm formation of *S. salivarius* strains was examined by the described method [57] with modifications [56]. Overnight cultures in biofilm medium (BM) [58] containing 20 mM glucose (BMG) were diluted at 1:20 in BMG and grown to an OD_600_ of 0.8. The culture was sonicated as described above, followed by dilution at 1:100 in BMG. The diluted suspension was then inoculated in the wells of a 96-well microtiter plate. The plate was incubated at 37 °C in 10% CO_2_ for 24 h. The biofilm was stained with crystal violet and the absorbance at 562 (OD_562_) nm was determined with a microplate reader (SoftMax^®^Pro, Molecular Devices).

The biofilm grown in a flow-cell system was examined as described [56]. Overnight cultures in BMG were diluted at 1:20 and grown to an OD_600_ of 0.8. The cultures were diluted at 1:800 in BMG and 300 μl of the diluted cell suspension was injected into the flow cell chamber. The chamber was kept at 37 °C in an upside down position without medium flow for 4 h. The medium flow rate was set up at 5 ml h^− 1^ channel^− 1^ and the biofilm was grown for 10 h. The biofilm was stained with SYTO 9/propidium iodide (PI) reagent (Invitrogen) and observed by using confocal laser scanning microscope (CLSM).

### The pH drop assay

The glycolytic activity of *S. salivarius* 57.I and its derivatives was evaluated by the acid drop analysis [59]. Cultures at an OD_600_ of 0.6 in BHI were harvested, washed once with 50 mM KCl-1 mM MgCl_2_, and concentrated in the same solution with an OD_600_ of 1.0. The cell suspension was equilibrated at pH 7.2 with 0.01 N KOH at room temperature, and the pH drop was initiated with the addition of 55.5 mM glucose. The pH value was recorded every 30 s for 1 h.

### Assays for acid resistance and oxidative stress tolerance

The growth of *S. salivarius* 57.I and it derivatives at pH 5.5 was evaluated using the described method [11] with modifications. Overnight cultures were diluted at 1:20 in BHI and grown to an OD_600_ of 0.8. *S. salivarius* Δ*codY* was subject to sonication three times prior to reading. The cultures were serially diluted and 10 μl of each dilution was spotted on BHI agar (pH 7.2) or BHI agar that has been adjusted to pH 5.5 by the addition of 2 N HCl (BHI-HCl). The plates were incubated at 37 °C in 10% CO_2_ for 24 h.

A similar approach was use to evaluate oxidative stress tolerance. Cultures were prepared as described above, serially diluted and spotted onto BHI agar plates supplemented with 0.5, 1, 2 or 3 mM H_2_O_2_. Plates were incubated at 37 °C in 10% CO_2_ for 24 h.

### *Galleria mellonella* larva model

The toxicity of *S. salivarius* strains in the *G. mellonella* larva model was determined using the described method [60] with modifications. Bacterial cells were grown to an OD_600_ of 0.8 in BHI, harvested, washed twice with PBS, and suspended in PBS at 1.0 × 10^9^ CFU ml^− 1^. 10 μl of the suspension was injected into the proleg of each larva with a 26 s ga needle (Hamilton). PBS and heat inactivated (75 °C for 30 min) bacterial suspensions with the same CFU were used as negative controls. For each bacterial strain, 12 larvae were infected. The infected larvae were kept at 37 °C without feeding and recorded daily until the death of all larvae. The significant difference between infection groups was analyzed by using Kaplan Meier curve with log-rank test.

To determine the CFU in a larva, the larva was surface-cleaned with 70% ethanol, placed in a 2-ml microfuge tube containing 1 ml PBS and 12 zirconia/silica beads (2.3 mm diameter). The larva was homogenized in a Mini-Beadbeater (Biospec Products) for a total of 40 s at room temperature. The bacteria count in the homogenate was determined by serial dilutions and plating.

## Additional files

Additional file 1: **Figure S1.** The CodY binding consensus sequences in Gram positive bacteria. (A) The binding consensus derived from streptococcal species. A search for the consensus of the CodY binding motifs from 12 streptococcal species was conducted using the MEME suites. (B) An alignment of the CodY binding consensus from *Streptococcus* spp., *Lactococcus lactis*, *Bacillus* spp., *C. difficile* and *S. aureus*. N, A or C or G or T; R, A or G; W, A or T; Y, C or T. (PPTX 59 kb)

Additional file 2: **Table S1.** The RPKM values of the direct targets of CodY in *S. salivarius* 57.I and Δ*codY.* **Table S2.** The RPKM values of the indirect targets of CodY in *S. salivarius* 57.I and Δ*codY.* **Table S3.** The RPKM values of genes encoding enzymes of the EMP pathway in *S. salivarius* 57.I and Δ*codY.* **Table S4.** Bacterial strains used in this study. **Table S5.** Oligonucleotides used in this study. (DOCX 30 kb)

## Abbreviations

BCAAs: Branched-chain amino acids
BHI: Brain heart infusion medium
BMG: Biofilm medium containing 20 mM glucose
CLSM: Confocal laser scanning microscope
FBP: Fibronectin-binding protein
GDH: Glutamate dehydrogenase
PI: Propidium iodide
PWM: Position weight matrix
RPKM: Reads per Kb per million mapped reads
TY: Tryptone-yeast extract medium
