# Supplementary material for: Impact of growth pH and glucose concentrations on the CodY regulatory network in Streptococcus salivarius
Source: BMC Genomics. 2018 May 23;19:386. doi: 10.1186/s12864-018-4781-z (PMC5966866; doi:10.1186/s12864-018-4781-z)
Supplement: Supplementary file 1 — Figure S1. The CodY binding consensus sequences in Gram positive bacteria. (A) The binding consensus derived from streptococcal species. A search for the consensus of the CodY binding motifs from 12 streptococcal species was conducted using the MEME suites. (B) An alignment of the CodY binding consensus from Streptococcus spp., Lactococcus lactis, Bacillus spp., C. difficile and S. aureus. N, A or C or G or T; R, A or G; W, A or T; Y, C or T. (PPTX 59 kb) [file 12864_2018_4781_MOESM1_ESM.pptx]

## Slide 1
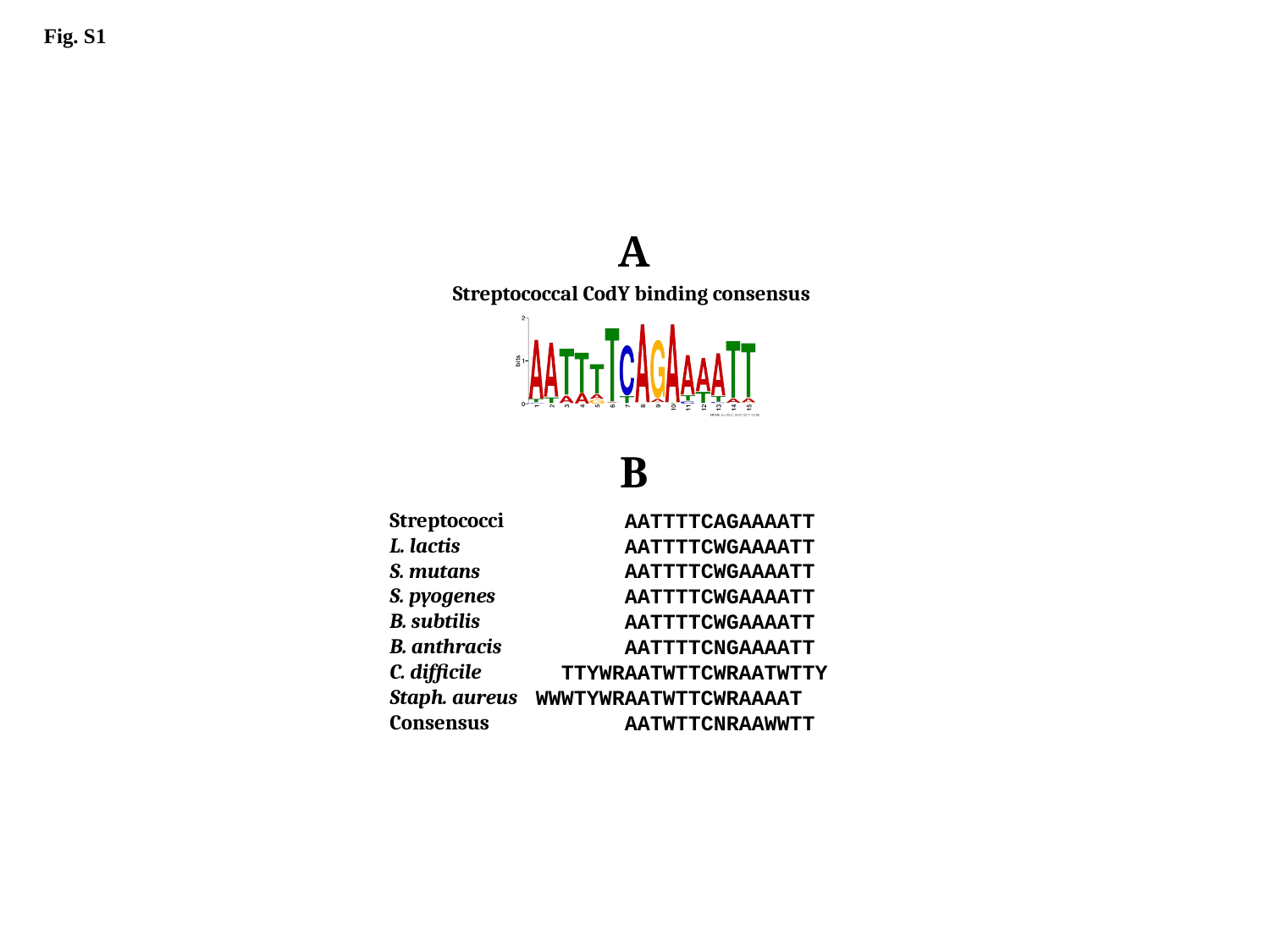

Fig. S1
A
Streptococcal CodY binding consensus
B
 AATTTTCAGAAAATT
 AATTTTCWGAAAATT
 AATTTTCWGAAAATT
 AATTTTCWGAAAATT
 AATTTTCWGAAAATT
 AATTTTCNGAAAATT
 TTYWRAATWTTCWRAATWTTY
WWWTYWRAATWTTCWRAAAAT
 AATWTTCNRAAWWTT
Streptococci
L. lactis
S. mutans
S. pyogenes
B. subtilis
B. anthracis
C. difficile
Staph. aureus
Consensus
