# Supplementary material for: Impact of growth pH and glucose concentrations on the CodY regulatory network in Streptococcus salivarius
Source: BMC Genomics. 2018 May 23;19:386. doi: 10.1186/s12864-018-4781-z (PMC5966866; doi:10.1186/s12864-018-4781-z)
Supplement: Supplementary file 2 — Table S1. The RPKM values of the direct targets of CodY in S. salivarius 57.I and ΔcodY. Table S2. The RPKM values of the indirect targets of CodY in S. salivarius 57.I and ΔcodY. Table S3. The RPKM values of genes encoding enzymes of the EMP pathway in S. salivarius 57.I and ΔcodY. Table S4. Bacterial strains used in this study. Table S5. Oligonucleotides used in this study. (DOCX 30 kb) [file 12864_2018_4781_MOESM2_ESM.docx]

TABLE S1. The RPKM values of the direct targets of CodY in *S. salivarius* 57.I and Δ*codY*

| **Strain** | *S. salivarius* 57.I^a^ | | | | *S. salivarius* Δ*codY*^a^ | | | |
| --- | --- | --- | --- | --- | --- | --- | --- | --- |
| **Growth condition** | Glu20/  pH 7 | Glu20/  pH 5.5 | Glu100/  pH 7 | Glu100/  pH 5.5 | Glu20/  pH 7 | Glu20/  pH 5.5 | Glu100/  pH 7 | Glu100/  pH 5.5 |
| **Locus** |  |  |  |  |  |  |  |  |
| Ssal_00190 | 1072 | 847 | 2104 | 959 | 1207 | 1288 | 3153 | 1121 |
| Ssal_00280 | 78 | 74 | 59 | 66 | 80 | 316 | 72 | 84 |
| Ssal_01946 | 380 | 1267 | 370 | 990 | 1270 | 1280 | 372 | 676 |
| Ssal_00131 | 63 | 140 | 430 | 322 | 200 | 1076 | 467 | 994 |
| Ssal_00132 | 451 | 963 | 52 | 1256 | 1646 | 3807 | 4574 | 6425 |
| Ssal_00133 | 797 | 829 | 629 | 1428 | 4026 | 5181 | 2752 | 3446 |
| Ssal_00134 | 615 | 636 | 915 | 1835 | 3281 | 6162 | 2180 | 2915 |
| Ssal_01037 | 124 | 261 | 287 | 480 | 453 | 1390 | 506 | 244 |
| Ssal_01567 | 115 | 145 | 163 | 534 | 661 | 522 | 1803 | 2233 |
| Ssal_02023 | 71 | 131 | 459 | 257 | 222 | 437 | 494 | 446 |
| Ssal_01828 | 86 | 111 | 259 | 373 | 146 | 476 | 582 | 636 |
| Ssal_01829 | 87 | 110 | 267 | 372 | 130 | 741 | 1147 | 654 |
| Ssal_01830 | 56 | 59 | 11 | 210 | 77 | 208 | 405 | 265 |
| Ssal_01831 | 49 | 32 | 108 | 173 | 70 | 298 | 371 | 446 |
| Ssal_01832 | 64 | 50 | 135 | 259 | 85 | 622 | 749 | 512 |
| Ssal_01272 | 372 | 555 | 1104 | 589 | 672 | 584 | 757 | 391 |
| Ssal_01273 | 208 | 217 | 317 | 522 | 493 | 663 | 687 | 464 |
| Ssal_01275 | 148 | 165 | 19 | 405 | 413 | 457 | 389 | 371 |
| Ssal_01276 | 151 | 158 | 222 | 352 | 459 | 692 | 406 | 390 |
| Ssal_00127 | 229 | 261 | 395 | 558 | 357 | 263 | 1143 | 807 |
| Ssal_00456 | 23 | 59 | 138 | 79 | 127 | 75 | 75 | 130 |
| Ssal_00457 | 46 | 84 | 104 | 166 | 68 | 206 | 87 | 264 |
| Ssal_00458 | 39 | 62 | 82 | 120 | 33 | 218 | 44 | 67 |
| Ssal_00459 | 56 | 111 | 82 | 111 | 41 | 253 | 74 | 175 |
| Ssal_00461 | 59 | 94 | 271 | 147 | 49 | 227 | 75 | 102 |
| Ssal_00462 | 36 | 87 | 71 | 84 | 18 | 184 | 51 | 63 |
| Ssal_00464 | 55 | 151 | 107 | 130 | 51 | 411 | 47 | 78 |
| Ssal_00465 | 91 | 274 | 151 | 156 | 101 | 599 | 200 | 232 |
| Ssal_00537 | 109 | 167 | 134 | 226 | 223 | 428 | 339 | 596 |
| Ssal_00538 | 80 | 107 | 156 | 261 | 118 | 539 | 355 | 445 |
| Ssal_00539 | 125 | 124 | 202 | 332 | 389 | 801 | 338 | 858 |
| Ssal_00884 | 73 | 93 | 103 | 111 | 135 | 124 | 166 | 122 |
| Ssal_00898 | 5 | 10 | 62 | 47 | 12 | 34 | 172 | 178 |
| Ssal_00899 | 6 | 12 | 101 | 77 | 28 | 55 | 280 | 256 |
| Ssal_00900 | 7 | 16 | 90 | 65 | 29 | 40 | 388 | 200 |
| Ssal_01351 | 78 | 143 | 90 | 156 | 143 | 417 | 315 | 249 |
| Ssal_01352 | 43 | 64 | 6 | 68 | 45 | 173 | 147 | 77 |
| Ssal_01655 | 23 | 88 | 118 | 67 | 121 | 201 | 256 | 266 |
| Ssal_01693 | 217 | 374 | 659 | 327 | 547 | 588 | 328 | 549 |
| Ssal_01694 | 276 | 268 | 643 | 579 | 507 | 490 | 390 | 598 |
| Ssal_01811 | 51 | 155 | 77 | 112 | 304 | 800 | 722 | 629 |
| Ssal_01812 | 231 | 380 | 2294 | 1286 | 759 | 813 | 1631 | 2238 |
| Ssal_01840 | 19 | 47 | 24 | 111 | 19 | 26 | 120 | 104 |
| Ssal_01891 | 63 | 335 | 269 | 891 | 502 | 2783 | 626 | 830 |
| Ssal_01892 | 52 | 160 | 178 | 427 | 182 | 798 | 422 | 785 |
| Ssal_01894 | 41 | 203 | 139 | 307 | 81 | 663 | 302 | 503 |
| Ssal_01895 | 27 | 81 | 55 | 159 | 32 | 508 | 96 | 119 |
| Ssal_01896 | 31 | 93 | 56 | 175 | 27 | 325 | 257 | 149 |
| Ssal_01897 | 39 | 98 | 89 | 258 | 53 | 425 | 175 | 201 |
| Ssal_01898 | 61 | 276 | 117 | 305 | 46 | 1052 | 344 | 378 |
| Ssal_01900 | 92 | 412 | 1183 | 882 | 382 | 1027 | 616 | 2324 |
| Ssal_01901 | 49 | 244 | 102 | 456 | 53 | 282 | 190 | 514 |
| Ssal_01902 | 63 | 170 | 105 | 596 | 39 | 483 | 187 | 293 |
| Ssal_01903 | 41 | 203 | 127 | 594 | 79 | 701 | 275 | 934 |
| Ssal_01904 | 4 | 42 | 3 | 7 | 25 | 85 | 36 | 29 |
| Ssal_00845 | 4 | 8 | 11 | 36 | 9 | 15 | 633 | 499 |
| Ssal_00908 | 24 | 17 | 46 | 120 | 821 | 287 | 2541 | 1988 |
| Ssal_01154 | 3 | 5 | 18 | 34 | 4 | 15 | 138 | 44 |
| Ssal_01464 | 38 | 29 | 94 | 136 | 270 | 203 | 668 | 330 |
| Ssal_01465 | 11 | 22 | 100 | 65 | 55 | 152 | 270 | 160 |
| Ssal_01466 | 18 | 17 | 107 | 66 | 153 | 134 | 421 | 315 |
| Ssal_01667 | 3 | 22 | 67 | 89 | 130 | 181 | 224 | 530 |
| Ssal_01668 | 7 | 14 | 4 | 65 | 112 | 279 | 232 | 485 |
| Ssal_00404 | 358 | 654 | 1020 | 903 | 0 | 0 | 0 | 0 |
| Ssal_00962 | 72 | 55 | 57 | 84 | 1559 | 1029 | 1237 | 1664 |
| Ssal_01704 | 8 | 37 | 83 | 72 | 20 | 26 | 757 | 147 |
| Ssal_01769 | 3 | 14 | 9 | 9 | 4 | 9 | 44 | 12 |
| Ssal_00555 | 43 | 114 | 91 | 286 | 96 | 63 | 188 | 414 |
| Ssal_00556 | 37 | 112 | 60 | 224 | 59 | 57 | 191 | 375 |
| Ssal_01768 | 384 | 637 | 2331 | 2014 | 4946 | 10010 | 8175 | 12638 |
| Ssal_01359 | 88 | 91 | 126 | 134 | 214 | 283 | 514 | 451 |
| Ssal_01360 | 2132 | 1175 | 2022 | 4538 | 7256 | 7419 | 2474 | 6651 |
| Ssal_01545 | 67 | 68 | 133 | 74 | 312 | 186 | 434 | 225 |
| Ssal_01546 | 45 | 43 | 105 | 65 | 173 | 212 | 316 | 210 |
| Ssal_01810 | 65 | 124 | 59 | 159 | 54 | 103 | 212 | 115 |
| Ssal_00112 | 68 | 97 | 137 | 258 | 235 | 222 | 608 | 371 |
| Ssal_01429 | 159 | 167 | 209 | 176 | 116 | 541 | 168 | 214 |
| Ssal_00672 | 25 | 20 | 28 | 35 | 1794 | 4909 | 2063 | 1479 |
| Ssal_00714 | 269 | 108 | 172 | 215 | 267 | 790 | 376 | 162 |
| Ssal_01428 | 241 | 155 | 508 | 137 | 128 | 601 | 613 | 1192 |
| Ssal_00214 | 162 | 469 | 88 | 1702 | 126 | 448 | 58 | 3492 |
| Ssal_00216 | 43 | 50 | 36 | 327 | 140 | 106 | 100 | 147 |
| Ssal_01153 | 100 | 115 | 398 | 142 | 188 | 120 | 383 | 581 |

^a^, Chemostat cultures grown at pH 7 or 5.5, with 20 mM (Glu20) or 100 mM (Glu100) glucose.

TABLE S2. The RPKM values of the indirect targets of CodY in *S. salivarius* 57.I and Δ*codY*

| **Strain** | *S. salivarius* 57.I^a^ | | | | *S. salivarius* Δ*codY*^a^ | | | |
| --- | --- | --- | --- | --- | --- | --- | --- | --- |
| **Growth condition** | Glu20/  pH 7 | Glu20/  pH 5.5 | Glu100/  pH 7 | Glu100/  pH 5.5 | Glu20/  pH 7 | Glu20/  pH 5.5 | Glu100/  pH 7 | Glu100/  pH 5.5 |
| **Locus** |  |  |  |  |  |  |  |  |
| Ssal_00236 | 1331 | 1027 | 2040 | 1533 | 10 | 5 | 23 | 17 |
| Ssal_00563 | 370 | 246 | 78 | 191 | 20 | 10 | 5 | 21 |
| Ssal_00678 | 12 | 18 | 36 | 42 | 0 | 7 | 8 | 3 |
| Ssal_00697 | 145 | 143 | 298 | 366 | 6 | 40 | 93 | 66 |
| Ssal_00723 | 35 | 149 | 272 | 183 | 1 | 1 | 31 | 10 |
| Ssal_00725 | 209 | 422 | 168 | 171 | 7 | 34 | 35 | 78 |
| Ssal_00726 | 279 | 552 | 325 | 188 | 116 | 84 | 96 | 220 |
| Ssal_00728 | 106 | 193 | 100 | 213 | 1 | 6 | 22 | 54 |
| Ssal_00729 | 122 | 257 | 56 | 45 | 0 | 11 | 7 | 9 |
| Ssal_00732 | 813 | 809 | 271 | 75 | 66 | 333 | 9 | 24 |
| Ssal_00733 | 657 | 694 | 213 | 37 | 9 | 323 | 3 | 5 |
| Ssal_00734 | 1959 | 2182 | 384 | 375 | 10 | 74 | 29 | 42 |
| Ssal_00735 | 1784 | 1599 | 231 | 193 | 2 | 49 | 25 | 29 |
| Ssal_00736 | 1298 | 1067 | 288 | 152 | 1 | 24 | 13 | 24 |
| Ssal_00737 | 924 | 972 | 293 | 85 | 0 | 29 | 10 | 11 |
| Ssal_00755 | 285 | 624 | 271 | 112 | 1 | 15 | 10 | 33 |
| Ssal_00854 | 3 | 13 | 25 | 15 | 0 | 5 | 5 | 0 |
| Ssal_00871 | 237 | 1120 | 1568 | 486 | 3 | 12 | 22 | 61 |
| Ssal_01340 | 4 | 5 | 28 | 15 | 0 | 1 | 1 | 3 |
| Ssal_01662 | 77 | 127 | 69 | 52 | 5 | 12 | 8 | 6 |
| Ssal_01837 | 449 | 2003 | 1951 | 1535 | 5 | 11 | 93 | 119 |
| Ssal_01861 | 108 | 431 | 759 | 600 | 19 | 108 | 233 | 135 |
| Ssal_00446 | 9 | 116 | 68 | 173 | 85 | 309 | 727 | 321 |
| Ssal_00630 | 52 | 91 | 184 | 56 | 98 | 203 | 317 | 194 |
| Ssal_00969 | 453 | 141 | 576 | 116 | 4015 | 2082 | 1378 | 629 |
| Ssal_01025 | 125 | 49 | 42 | 237 | 388 | 821 | 349 | 436 |
| Ssal_01086 | 27 | 36 | 24 | 46 | 68 | 137 | 82 | 85 |
| Ssal_01401 | 17 | 13 | 12 | 20 | 64 | 95 | 62 | 142 |
| Ssal_01479 | 40 | 49 | 3 | 6 | 152 | 282 | 63 | 31 |
| Ssal_01624 | 70 | 147 | 89 | 78 | 12466 | 5860 | 334 | 102 |
| Ssal_01876 | 1424 | 895 | 2348 | 1695 | 12466 | 5859 | 8532 | 9715 |
| Ssal_02041 | 1034 | 613 | 827 | 1902 | 1684 | 4420 | 2147 | 3105 |
| Ssal_02096 | 16 | 23 | 34 | 22 | 60 | 64 | 205 | 69 |
| Ssal_00088 | 2084 | 2884 | 3759 | 2136 | 79 | 1373 | 614 | 463 |
| Ssal_00221 | 162 | 348 | 1012 | 510 | 45 | 151 | 172 | 136 |
| Ssal_00318 | 116 | 527 | 650 | 233 | 16 | 128 | 233 | 43 |
| Ssal_00689 | 154 | 139 | 280 | 309 | 17 | 67 | 89 | 71 |
| Ssal_00700 | 104 | 169 | 634 | 347 | 6 | 59 | 89 | 36 |
| Ssal_00703 | 217 | 187 | 431 | 335 | 13 | 64 | 104 | 63 |
| Ssal_00750 | 41 | 37 | 260 | 49 | 14 | 17 | 56 | 16 |
| Ssal_00859 | 20 | 44 | 65 | 37 | 4 | 18 | 24 | 9 |
| Ssal_01403 | 14 | 95 | 15 | 182 | 0 | 2 | 4 | 4 |
| Ssal_01497 | 9 | 37 | 43 | 1638 | 3 | 9 | 16 | 92 |
| Ssal_01636 | 77 | 193 | 172 | 337 | 10 | 72 | 34 | 21 |
| Ssal_01796 | 1423 | 1851 | 3322 | 4496 | 325 | 990 | 465 | 707 |
| Ssal_01797 | 1440 | 1887 | 2643 | 3412 | 183 | 754 | 247 | 559 |
| Ssal_01798 | 1070 | 1233 | 131 | 3070 | 129 | 1038 | 172 | 603 |
| Ssal_01799 | 277 | 256 | 603 | 1081 | 31 | 275 | 31 | 80 |
| Ssal_01800 | 1842 | 1800 | 9108 | 5023 | 502 | 2159 | 444 | 1045 |
| Ssal_01801 | 1197 | 1537 | 2352 | 3675 | 502 | 1209 | 521 | 2500 |
| Ssal_01802 | 1354 | 1973 | 7870 | 4562 | 212 | 769 | 371 | 683 |
| Ssal_01803 | 925 | 1036 | 1487 | 2643 | 92 | 579 | 176 | 358 |
| Ssal_01804 | 844 | 1593 | 1651 | 3214 | 72 | 609 | 203 | 517 |
| Ssal_01805 | 611 | 661 | 3338 | 2104 | 38 | 544 | 119 | 344 |
| Ssal_01806 | 1507 | 1465 | 4075 | 2570 | 474 | 529 | 654 | 1364 |
| Ssal_01807 | 666 | 770 | 2514 | 1625 | 166 | 387 | 434 | 316 |
| Ssal_01838 | 19 | 121 | 129 | 95 | 4 | 9 | 19 | 18 |
| Ssal_02030 | 172 | 199 | 175 | 357 | 1 | 13 | 21 | 36 |
| Ssal_02089 | 102 | 213 | 364 | 198 | 27 | 92 | 38 | 32 |
| Ssal_00154 | 33 | 46 | 12 | 69 | 272 | 207 | 1050 | 183 |
| Ssal_00402 | 419 | 746 | 455 | 322 | 647 | 1477 | 1615 | 523 |
| Ssal_00485 | 10 | 64 | 91 | 56 | 44 | 339 | 598 | 154 |
| Ssal_00506 | 475 | 474 | 232 | 175 | 1054 | 1113 | 555 | 259 |
| Ssal_00717 | 130 | 92 | 181 | 171 | 785 | 2513 | 886 | 547 |
| Ssal_00665 | 22 | 89 | 67 | 94 | 79 | 199 | 157 | 273 |
| Ssal_00738 | 5 | 3 | 4 | 9 | 241 | 484 | 109 | 35 |
| Ssal_00740 | 40 | 13 | 37 | 6 | 1229 | 948 | 244 | 113 |
| Ssal_00742 | 105 | 69 | 139 | 72 | 381 | 916 | 247 | 89 |
| Ssal_00975 | 15 | 25 | 18 | 42 | 151 | 135 | 173 | 63 |
| Ssal_00982 | 63 | 74 | 96 | 78 | 119 | 138 | 160 | 95 |

^a^, Chemostat cultures grown at pH 7 or 5.5, with 20 mM (Glu20) or 100 mM (Glu100) glucose.

TABLE S3. The RPKM values of genes encoding enzymes of the EMP pathway in *S. salivarius* 57.I and Δ*codY*

| Locus | Gene | *S. salivarius* 57.I^a^ | | | | *S. salivarius* Δ*codY*^a^ | | | |
| --- | --- | --- | --- | --- | --- | --- | --- | --- | --- |
|  |  | Glu20/  pH 7 | Glu20/  pH 5.5 | Glu100/  pH 7 | Glu100/  pH 5.5 | Glu20/  pH 7 | Glu20/  pH 5.5 | Glu100/  pH 7 | Glu100/  pH 5.5 |
| Ssal_01383 | *glk* | 248 | 355 | 646 | 315 | 317 | 262 | 137 | 282 |
| Ssal_01270 | *pfk* | 1361 | 1733 | 2600 | 1287 | 516 | 1251 | 513 | 507 |
| Ssal_01268 | *pyk* | 1532 | 972 | 5383 | 2830 | 1059 | 2072 | 647 | 1085 |
| Ssal_00232 | *pgk* | 1023 | 990 | 1361 | 1100 | 490 | 1483 | 424 | 595 |

^a^, Chemostat cultures grown at pH 7 or pH 5.5, with 20 mM glucose (Glu20) or 100 mM glucose (Glu100)

TABLE S4. Bacterial strains used in this study

| ***S. salivarius* strains** | **Relevant phenotypes^a^** | **Description** | **Reference** |
| --- | --- | --- | --- |
| 57.I |  | Wild-type strain | 52 |
| Δ*codY* | Em^r^, CodY^-^ | Strain 57.I *codY*::*erm* | This study |
| CΔ*codY* | Em^r^, Km^r^, CodY^+^ | Strain Δ*codY* harboring Ω*kan*-*codY* at the intergenic region of Ssal_00777 and Ssal_00779 | This study |

^a^, r, resistant; -, inactivated; +, active; Em, erythromycin; Km, kanamycin.

TABLE S5. Oligonucleotides used in this study

| Oligonucleotide | Sequence^a^ |
| --- | --- |
| Ssal_00403_S | GGACAGCTTGTGTCAGTCTAG |
| Ssal_00405_AS | GCCCAATGGCAAAATCATGTGC |
| 57.I_CodY_800_XhoI_S | CTTCTCGAGCTGGACAAAAAGGCTTGTCC |
| 57.I_CodY_1900_SphI_AS | ATGCATGCGCCTTTGTCATATTACTTTCTCC |
| 57.I_lacZ_1241_S | GGGATATCAAAGTGATGAAAC |
| 57.I_lacZ_4970_BamHI_AS | ATCGGATCCAAATATATCGTTGGAGGCAAA |
| 57.I_lacZ_4970_SphI_S | TTTGCATGCGATAAAGGAAACTCGAAAGGG |
| 57.I_ssal_00779_5950_AS | GGTAGCCATCAATTCGTAACAGACTCCC |
| kan_BamHI_S | GTGGATCCTCGATAAGCTTGGATCC |
| kan_XhoI_AS | GCGCTCGAGGCTCTCCGGATCC |

^a^, Inserted restriction recognition sites are underlined.
